# Corrigendum to “Basal Activation of Astrocytic Nrf2 in Neuronal Culture Media: Challenges and Implications for Neuron-Astrocyte Modelling”

**DOI:** 10.1177/23982128261470929

**Published:** 2026-07-23

Elsharkasi MMO, Villani B, Wells G, et al. F. Basal activation of astrocytic Nrf2 in neuronal culture media: Challenges and implications for neuron-astrocyte modelling. *Brain and Neuroscience Advances*. 2025;9. doi:10.1177/23982128251351360

The authors would like to alert readers to the following changes in their article:

An error was made in the reporting of the astrocyte media components on page 2.

The ScienCell primary human cortical astrocytes were cultured in Complete Astrocyte Media (SC-1801) containing astrocyte growth supplement (AGS; 1% v/v; #SC-1852), foetal bovine serum (FBS; 2% v/v, #SC-0010) and Penicillin/Streptomycin (Pen/Strep; 1% v/v, #SC-0503), rather than the 5% AGS, 10% FBS and 5% Pen/Strep that we reported, as per the manufacturers’ instructions. We apologise for our oversight on this error in editing and hope that this clarifies the conditions that we used for astrocyte culture for informing future studies.

We are grateful for conversations with other researchers interested in our work which have brought this error to our attention.

The Journal Editor confirms that these changes do not affect the overall conclusions of this article.

