# Supplementary material for: Basal activation of astrocytic Nrf2 in neuronal culture media: Challenges and implications for neuron-astrocyte modelling
Source: Brain Neurosci Adv. 2025 Jul 24;9:23982128251351360. doi: 10.1177/23982128251351360 (PMC12290377; doi:10.1177/23982128251351360)
Supplement: sj-docx-1-bna-10.1177_23982128251351360 – Supplemental material for Basal activation of astrocytic Nrf2 in neuronal culture media: Challenges and implications for neuron-astrocyte modelling [file sj-docx-1-bna-10.1177_23982128251351360.docx]

**Basal activation of astrocytic Nrf2 in neuronal culture media: challenges and implications for neuron-astrocyte modelling**

**Supplementary Information**

**Supplementary Figures**

**
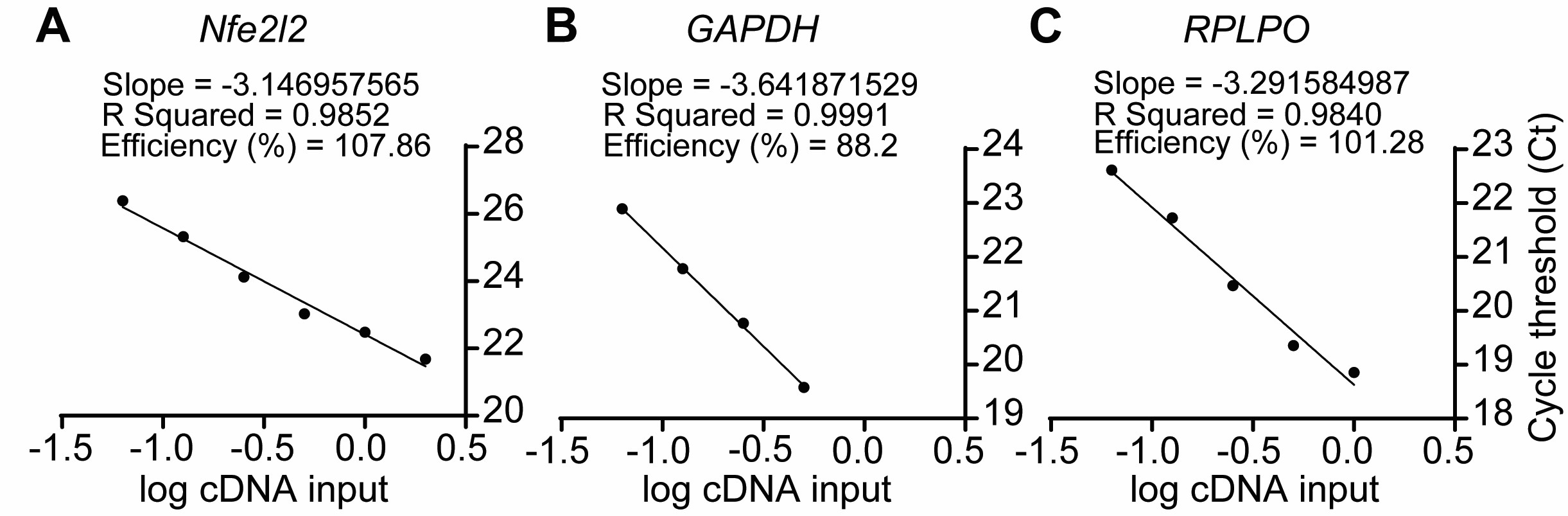
**

**Figure S1: Standard Curve optimisation of A. *Nfe2l2*, B. *GAPDH* and *C. RPLPO* primers.** cDNA from primary human astrocyte cultures was serially diluted, two-fold, over the arbitrary amounts depicted with neat cDNA representing a value of 1.0 (log value 0). qRT-PCR was performed with each primer pair using diluted cDNA and plotted as cycle thresholds (Ct) against log10 cDNA input amount. Slopes were extrapolated from each linear standard curve, and used to calculate efficiency values, using the formula Efficiency = (10e(-1/SLOPE)-1) x 100, and correlation coefficients (R^2^).


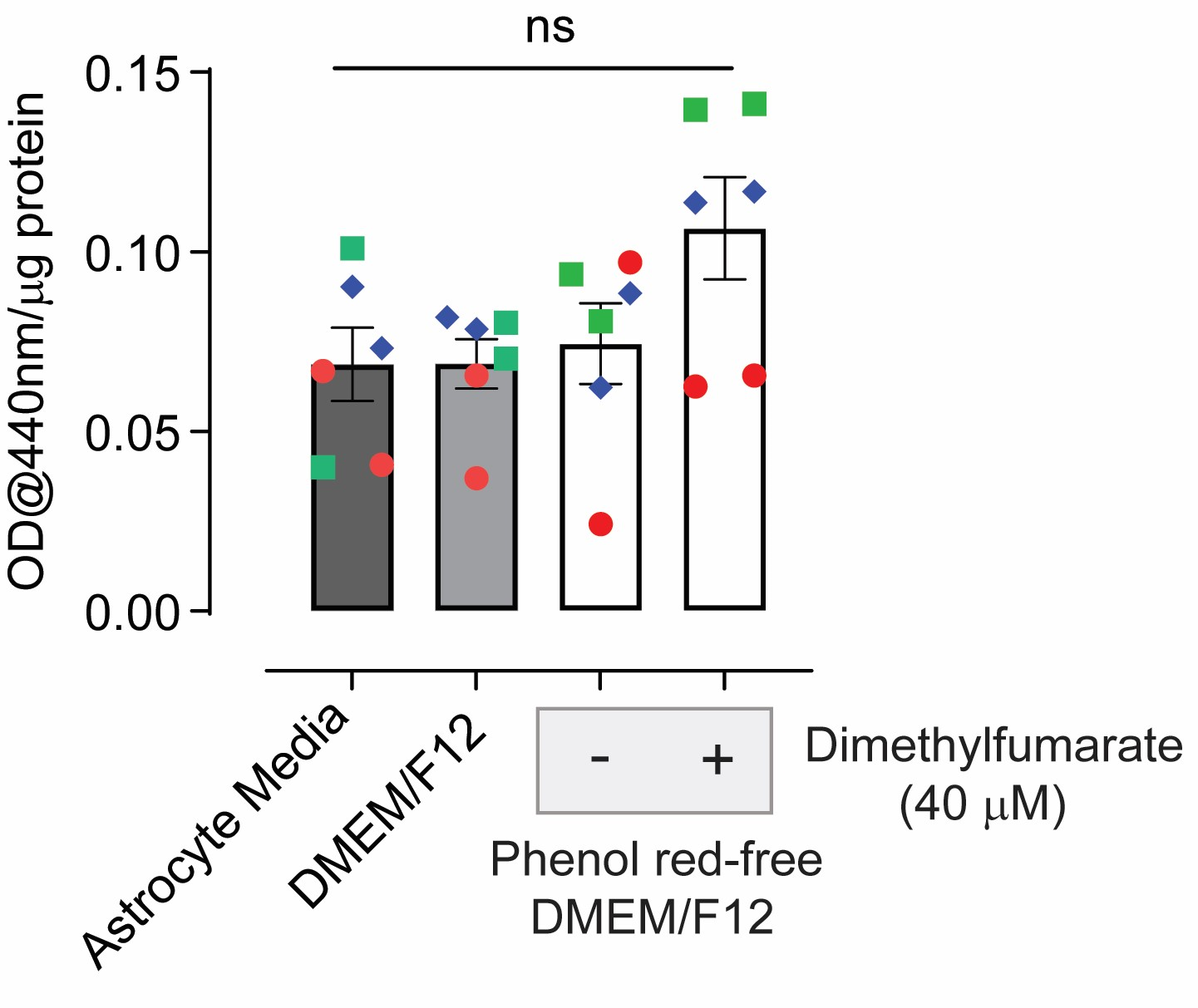


**Figure S2: Effects of phenol red on activation of astrocytic Nrf2.** Human astrocyte cultures were maintained in DMEM/F12 media with (Thermofisher #11320033) or without phenol red (Thermofisher #21041025) and Nrf2 activity measured via end-point measurement of NQO1 enzymatic activity. Effects of 40 µM DMF were also tested in phenol-red-free DMEM/F12. Data are presented as mean absorbance/ug total protein ± SEM (n=6, three independent experiments). Analysis in (B) was performed using One-way ANOVA (*P*=0.0703) followed by Tukey’s post-hoc analyses (*P*=0.1960 comparing phenol red-free DMEM/F12 + DMF to phenol red-free DMEM/F12 alone). Values derived from each independent vial of cells are denoted by colour.
